# Impact of previous cardiac function status assessed by echocardiography on the outcome of COVID-19

**DOI:** 10.1038/s41598-022-14887-3

**Published:** 2022-06-23

**Authors:** Irene Carrión, Carmen Olmos, María Luaces, Ana Isabel Cortés, Carlos Real, Alberto de Agustín, Roberta Bottino, Eduardo Pozo, Leopoldo Pérez de Isla, Fabián Islas

**Affiliations:** 1 Instituto Cardiovascular, Hospital Clínico San Carlos, Instituto de Investigación Sanitaria del Hospital Clínico San Carlos (IdSSC), Prof. Martín Lagos s/n, 28040 Madrid, Spain; 2 Unidad de Innovación, Hospital Clínico San Carlos, Instituto de Investigación Sanitaria del Hospital Clínico San Carlos (IdSSC), Madrid, Spain

**Keywords:** Cardiology, Health care, Medical imaging

## Abstract

More than 91,000 fatalities due to Coronavirus Disease 2019 (COVID-19) have occurred in Spain. Several factors are associated with increased mortality in this disease, including cardiovascular risk factors (CVRF). However, information on the cardiac function of patients prior to the onset of COVID-19 is scarce and the potential impact it may have is uncertain. The aim of the EchoVID study was to describe the potential association between CVRF and cardiac function status prior to SARS-CoV-2 infection and in-hospital mortality. We studied clinical characteristics and cardiac function of patients admitted during the first wave of COVID-19. All patients had a transthoracic echocardiogram performed in the previous 12 months prior to diagnosis; conventional systolic and diastolic function parameters were analyzed. Logistic regression analysis was performed to identify predictors of in-hospital mortality. We included 296 individuals. Median age was higher in the group of patients who died (81.0 vs 76.1 years; p = 0.007). No significant differences were found in CVRF. Survivors were more frequently receiving anticoagulation therapy (52.9% vs 70.8%; p = 0.003). LVEF, although preserved on average in both groups, was significantly lower in the group of deceased patients (56.9% vs 61.1%; p = 0.017). Average E/e′ ratio was higher in the deceased group (11.1 vs 10.1; p = 0.049). Five variables were found to be independently associated with in-hospital mortality due to COVID-19: Age, male gender, LVEF, E/e′ ratio and anticoagulation therapy. A model including these variables had an area under the ROC curve of 0.756 (CI 0.669–0.843). The echocardiographic variables included in the model significantly improved the discriminative power, compared to a model including only demographic data. Left ventricular ejection fraction and E/e′ ratio prior to SARS-CoV-2 infection are two easily-obtained echocardiographic parameters that provide additional prognostic information over clinical factors when assessing patients admitted for SARS-CoV-2 infection.

## Introduction

Up to January 2022 352,298,821 cases of Severe Acute Respiratory Syndrome Coronavirus 2 (SARS-CoV-2) infections and more than 5.5 million related deaths have been reported worldwide. More than 91,000 fatalities due to Coronavirus Disease 2019 (COVID-19) have occurred in Spain^1^. Several risk factors associated with increased mortality in this disease have been described, including cardiovascular risk factors (CVRFs) such as hypercholesterolemia and type 2 diabetes mellitus (DM)^2^.

COVID-19 disease primarily affects the respiratory system; however, many other organs can be affected, leading to systemic inflammation and sepsis. To date, several clinical studies describing deleterious cardiac consequences of COVID-19 documented by transthoracic echocardiography (TTE) have been published^3–6^. A variety of findings have been observed in these studies, among which left ventricular diastolic dysfunction and right ventricular systolic dysfunction stand out for their frequency^4–6^. However, information on the cardiac function of patients prior to the onset of COVID-19 is scarce and the potential impact it may have on their clinical course during hospital admission is unknown. Thus, the aim of the EchoVID study was to describe the potential association between CVRF and cardiac function status studied by a TTE performed prior to SARS-CoV-2 infection, and in-hospital mortality.

## Methods

A retrospective, observational and descriptive study was performed on patients admitted to a tertiary care university hospital between March and June 2020 at the time of the first wave COVID-19 health emergency. During this period, 1018 admitted patients diagnosed with COVID-19 either confirmed by positive PCR for SARS-CoV-2 or with high clinical suspicion, and who had been studied in the past with at least one TTE were included in a database. TTE was performed in the 12 months prior to admission in 296 of these patients, and they conformed our study cohort. Demographic, clinical (previous comorbidities, cardiovascular risk factors and treatment) and blood test data (lipid profile, renal function) were obtained from each patient's electronic medical record.

The hospital ethics committee (CEIC Hospital Clínico San Carlos) approved the implementation of the study and it was performed in accordance with the declaration of Helsinki. The use of informed consent was not necessary, since the use of patient data was anonymized; this was approved by the CEIC.

### Echocardiography

All echocardiograms were performed by expert Cardiovascular Imaging cardiologists. Left ventricular dimensions and volumes as well as systolic and diastolic function were measured following European quantification guidelines^7,8^. The left ventricular ejection fraction (LVEF) measured by the biplane method was the chosen parameter for classifying systolic function. As for diastolic function, mitral flow, including E and A waves, as well as E/A ratio, were included for analysis. In addition, medial and lateral mitral annulus velocities (e′) acquired with tissue Doppler imaging in the 4-chamber view in order to subsequently obtain the E/e′ ratio. Left atrial volume was measured at end systole with the biplane area-length method. Tricuspid annulus systolic excursion (TAPSE), lateral annulus velocity (S′) and fractional area change (FAC) were used to evaluate right ventricular (RV) systolic function in the studies in which it had been reported. The severity of mitral, aortic and tricuspid regurgitation was assessed following the European Society of Cardiology recommendations and classified as non-significant (mild and moderate grade II) or significant (moderate grade III and severe)^9^.

### Statistical analysis

Quantitative variables were expressed as median and interquartile range (IQR). Assessment of normality and equality of variances for continuous data was performed using the Shapiro–Wilk test and the Levene test, respectively. As most continuous variables followed a non-normal distribution, the Mann–Whitney *U* test was used for comparison. Categorical variables were expressed as percentages with a 95% confidence interval (95% CI). Hypothesis testing was performed with Pearson's Chi-squared test and Fisher’s exact test when appropriate.

Logistic regression analysis was performed to identify the predictors of in-hospital mortality. Variables that were statistically significant in the univariable analysis (p < 0.05), or considered clinically relevant, were integrated in a multivariable regression model.

The final model was built by means of stepwise forward selection and backward elimination technique. The significance levels for selection and elimination were < 0.05 and ≥ 0.10, respectively. To control for confounding, we compared the estimated parameters of the full model with those of the final selected model. No difference between the estimated parameters exceeded 10%. The adjusted odds ratios (ORs) with 95% confidence intervals (CIs) for each variable finally included in the model were calculated.

In order to facilitate the applicability of the model, a second simplified model using literature-based cut-off values^7,8^ for the echocardiographic continuous variables included in the model (LVEF lower than 60% and E/e′ ratio higher than 14) was also constructed.

Performance of both final predictive models to predict in-hospital death was assessed by analyzing discrimination (receiver operating characteristic (ROC) curve) and goodness of fit (Hosmer–Lemeshow test, Cox–Snell and Nagelkerke R2 values). No significant multicollinearity (assessed using variance inflation factors) was detected.

Finally, we compared the predictive performance of our model with that of a model which included only demographic variables (gender and age). Differences in the discriminative power among models were assessed by comparing their ROC curves.

The significance level was set at a bilateral p < 0.05 value. All statistical analyses were conducted using Stata 15 (StataCorp, College Station, TX, USA).

## Results

A total of 1018 patients were admitted to our hospital from March to June 2020 with confirmed diagnosis of COVID-19 infection either with a positive PCR or high clinical suspicion of infection despite a negative PCR for the virus. Out of this total, 296 had at least one TTE performed during the previous 12 months, and they conformed our observational cohort. The mean time from TTE to admission was 5.3 ± 3.5 months.

Median age of the cohort was 77.9 (17.4) years and 57.8% of the patients were male. The prevalence of arterial hypertension (HT), type 2 diabetes mellitus (DM), dyslipidemia and addiction to tobacco was 35.8%, 19.6%, 41.2% and 22.0%, respectively. Only 15 patients were admitted to the ICU. Out of 296 patients included in the cohort, 87 died during admission (29.4%).

### Comparison of clinical and imaging features between survivor and non-survivor group

Main differences in baseline characteristics and echocardiographic values between the survivor group (n = 209) and the non-survivor group (n = 87) are depicted in Table 1.

**Table 1:** Baseline characteristics and echocardiographic values between the group of patients who died and the group of patients who survived SARS-CoV-2 infection.

	Total (n = 296)	Deceased (n = 87)	Survivors (n = 209)	p
**Demographic characteristics**				
Age (years)	77.9 (17.4)	81.0 (16.1)	76.1 (18.8)	**0.007**
Male gender (%)	57. 8	54.0	59.3	0.400
**Cardiovascular risk factors and comorbidities**				
HT (%)	35. 8	34.5	36.4	0.758
DM (%)	19.6	17.2	20.6	0.510
Dyslipidemia (%)	41.2	41.4	41.1	0.971
Obesity (%)	27.4	26.4	27.8	0.817
Tobacco (%)	22.0	19.5	23.0	0.517
AF (%)	25.7	26.4	25.4	0.847
IHD (%)	17.6	16.1	18.2	0.667
HF (%)	19.9	20.7	19.6	0.833
Stroke (%)	1.7	1.1	1.9	0.642
GFR (ml/min/1.73 m^2^)	65.0 (44.5)	53.2 (40.2)	72.2 (46)	**0.048**
**Cardiovascular drugs**				
Statin (%)	21.3	13.8	24.4	**0.045**
Beta-blocker (%)	29.1	25.3	30.6	0.357
ACEI (%)	15.9	13.8	16.7	0.527
ARA-2 (%)	9.8	14.9	7.7	0.055
Calcium antagonist (%)	23.6	17.2	26.3	0.094
**Anticoagulation**				
Anticoagulation therapy (%)	65.5	52.9	70.8	**0.003**
LMWH (%)	61.1	50.6	65.6	**0.016**
Anti-vitamin K (%)	9.1	3.45	11.5	**0.029**
DOAC (%)	8.1	4.6	9.6	0.153
**Echo parameters**				
Indexed LV mass (g/m^2^)	99.8 (41.0)	99.6 (45.2)	100.0 (39.9)	0.936
LVTDD (mm)	45.3 (9.5)	43.4 (10.4)	45.6 (8.5)	**0.059**
E/A	0.76 (0.4)	0.69 (0.3)	0.81 (0.4)	**0.007**
Mean E/e′	10.3 (5.0)	11.1 (5.6)	10.1 (4.3)	**0.049**
LVEF (%)	60.1 (11.7)	56.9 (11.9)	61.1 (10.7)	**0.017**
RVFAC (%)	44.5 (9)	44.0 (4)	45.0 (12)	0.767
S′ (cm/s)	11.5 (4.3)	11.5 (4.4)	11.5 (4.0)	0.754
TAPSE (mm)	20.6 (6.2)	20.3 (6.3)	20.9 (6.2)	0.209
PASP (mmHg)	23.4 (25.2)	25.7 (11.3)	23.2 (16.1)	0.727
Significant MR (%)	10.5	8.6	14.9	0.096
Significant AR (%)	7.4	6.7	9.2	0.457
Significant TR (%)	9.5	9.1	10.3	0.738

Values are presented as median and IQR or number and percentage. Bold values denote statistical significance at the p < 0.05 level. *ACEI* angiotensin-converting enzyme inhibitor, *AF* atrial fibrillation, *AR* aortic regurgitation, *ARA-2* aldosterone receptor 2 antagonist, *CKD* chronic kidney disease, *CVD* cerebrovascular disease, *DM* type 2 diabetes mellitus, *DOAC* direct-acting oral anticoagulant, *FAC* fractional area change, *HF* heart failure, *HT* hypertension, *IHD* ischemic heart disease, *LMWH* low-molecular-weight heparin, *LV* left ventricle, *LVEF* LV ejection fraction, *LVTDD* LV diastolic diameter, *MR* mitral regurgitation, *TAPSE* tricuspid annular plane systolic excursion, *TR* tricuspid regurgitation.

Median age was significantly higher in the group of patients who died (81.0 vs 76.1 years; p = 0.007). No statistically significant differences were identified between the groups in terms of gender or in the prevalence of cardiovascular risk factors such as HT, obesity, dyslipidemia, DM or smoking. Of note, a significant difference was found in the median glomerular filtration rate (GFR) at admission according to the MDRD formula (53.2 vs 72.2 ml/min/1.73 m^2^; p = 0.048).

The prevalence of atrial fibrillation, heart failure prior to admission and ischemic heart disease was similar in both groups.

Regarding the treatments followed by patients prior to admission, it is remarkable that more statins were taken in the survivor group (p = 0.045). In terms of anticoagulation, it is interesting to note that survivors were more frequently treated chronically with low molecular weight heparin (p = 0.016) and with vitamin K antagonists (p = 0.029). The prior use of direct-acting oral anticoagulants (DOACs), which was not very common in our groups (less than 10%), was not significantly different between them (p = 0.153). As for drugs acting on the Renin–Angiotensin–Aldosterone-System for both groups, there were no significant differences in previous treatment with an angiotensin-converting enzyme inhibitor (ACEI) or an aldosterone receptor 2 antagonist (ARA-2) (p = 0.527 and p = 0.055, respectively).

The percentage of patients admitted to the ICU was very low, and similar in both groups (6.9% vs 4.3%; p = 0.355).

When comparing echocardiographic parameters between the two groups, no significant differences were observed in indexed left ventricular (LV) mass, LV end-systolic volume or LV diameters.

**Figure 1:**
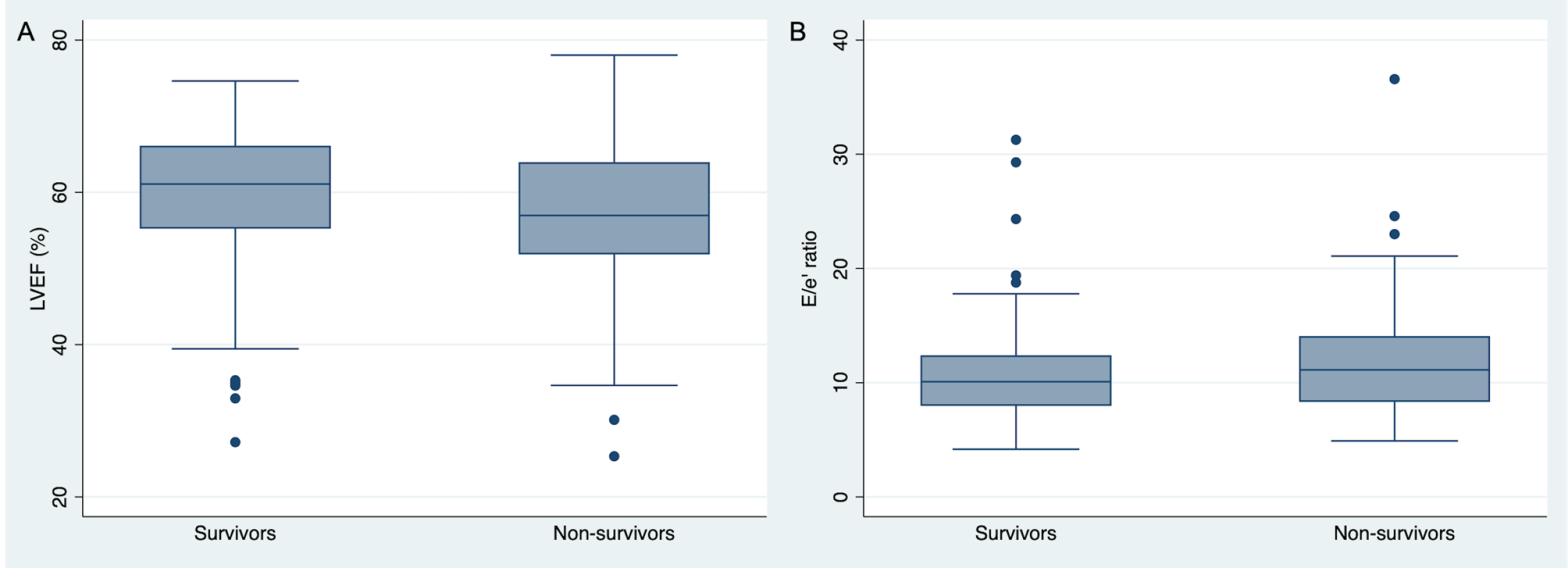
Differences in LVEF and E/e′ between survivor and non-survivor patients with SARS-CoV-2 infection. LVEF (**A**) and E/e′ ratio (**B**) distribution as box plots in survivor and non-survivor patients with SARS-CoV-2 infection. *LVEF* left ventricular ejection fraction.

With regard to systolic function, LVEF, although preserved on average in both groups, was significantly lower in the group of deceased patients (56.9% vs 61.1%; p = 0.017) (Fig. 1). Other parameters of LV systolic function such as global longitudinal strain or mitral S′ wave showed no differences, although it was only possible to quantify them in a limited number of patients.

Statistically significant differences were found in diastolic function parameters, more specifically the E/A ratio of mitral flow was lower in the group of deceased patients. Furthermore, the average E/e′ ratio was significantly higher in the deceased group (11.1 vs 10.1; p = 0.049) (Fig. 1). No significant differences appeared in RV function parameters between the two groups (TAPSE, S′ or FAC) or in the prevalence of significant valve disease.

### Clinical and echocardiographic predictors of in-hospital mortality

Variables significantly associated with in-hospital mortality in the univariable analysis and those considered clinically relevant were included in a multivariable logistic regression analysis: age, gender, GFR at admission, LVEF, E/e′ ratio, chronic treatment with statins and anticoagulation therapy (Table 2).

**Table 2:** Univariable and multivariable analysis for in-hospital mortality in hospitalized patients with SARS-CoV-2 infection.

	Univariable analysis		Multivariable analysis	
	OR (CI 95%)	p	OR (CI 95%)	p
Male gender	1.24 (0.75–2.05)	0.400	2.30 (1.06–4.99)	**0.034**
Age (years)	1.03 (1.00–1.06)	**0.021**	1.04 (1.00–1.08)	**0.079**
LVEF %	0.96 (0.94–0.99)	**0.028**	0.95 (0.91–0.98)	**0.011**
E/e′	1.10 (1.03–1.18)	**0.004**	1.09 (1.00–1.19)	**0.042**
Anticoagulation therapy	0.46 (0.28–0.77)	**0.003**	0.50 (0.23–1–07)	**0.075**
Statins	0.49 (0.25–0.98)	**0.045**	0.78 (0.30–2.05)	0.621
GFR (ml/min/1.73 m^2^)	0.99 (0.98–1.00)	**0.096**	0.96 (0.98–1.01)	0.595

Values are presented as odds ratio and 95% confidence intervals. Bold values denote statistical significance at the p < 0.10 level. *GFR* glomerular filtration rate, *LVEF* LV ejection fraction.

The five variables included in the final model were: male gender, age, LVEF, E/e′ and anticoagulation therapy.

The 5 variables independently associated with in-hospital mortality that were included in our final predictive EchoVID model were: age, male gender, LVEF, E/e′, and anticoagulation therapy. The model showed an area under the ROC curve of 0.752 (CI 0.666–0.837). The goodness of fit was satisfactory (Hosmer–Lemeshow test p = 0.654, Cox–Snell R2 0.151, Nagelkerke R2 0.216). The model had a 58% sensitivity and 87% specificity for predicting in-hospital mortality in our cohort.

The simplified 5-variable EchoVID model using literature-based cut-off values for the echocardiographic continuous variables of the model included the following variables: age, male gender, LVEF < 60%, E/e′ ratio higher > 14, anticoagulation status. The area under the ROC curve of this simplified model was 0.756 (CI 0.669–0.843). The goodness of fit was also satisfactory (Hosmer–Lemeshow test p = 0.190, Cox–Snell R2 0.139, Nagelkerke R2 0.220).

**Figure 2:**
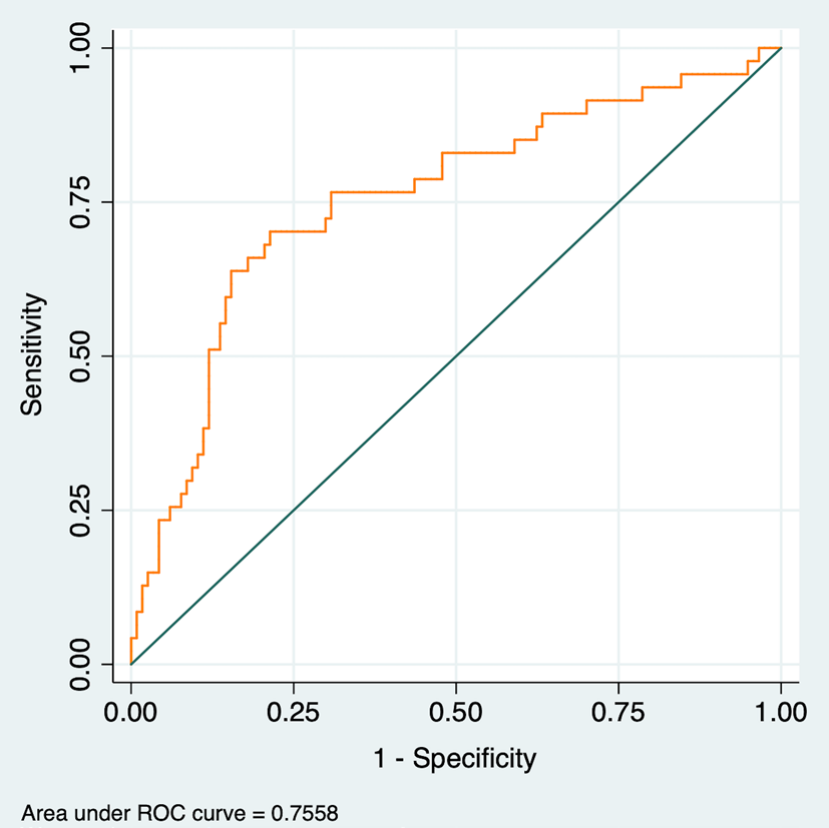
Receiver-operator characteristic (ROC) curves for our logistic regression model to predict in-hospital mortality. The model included the following variables: age, gender, left ventricular ejection fraction, E/e′ ratio and anticoagulation status.

The addition of the two echocardiographic parameters (LVEF and E/e′ ratio) to a model including only demographic date (age and gender) significantly improved the discriminative power for in-hospital mortality (p < 0.001). The final model with the two echocardiographic parameters had also a statistically significant superior predictive performance (p < 0.001), when compared with the model including only age, gender, and anticoagulation therapy (Table 3, Fig. 2).

**Table 3:** Comparison of the discriminative performance of different models for predicting in-hospital mortality in patients with SARS-CoV-2 infection.

	AUC	CI 95%
Model 1 (age + gender)	0.653	0.561–0.745
Model 2 (age + gender + anticoagulation)	0.671	0.580–0.762
EchoVID model (age + gender + anticoagulation + LVEF + E/e′)	0.756	0.669–0.843

Values are presented as area under the curve (AUC) and 95% confidence intervals (CI).

## Discussion

The results of this study show that cardiac function may have a determinant role in the outcome of patients affected by COVID-19; simple and feasible parameters such as LVEF and E/e′ provide prognostic information that could improve the initial risk assessment of patients.

We retrospectively analyzed 296 patients diagnosed with COVID-19. During in-hospital follow-up, almost 30% of patients died. Non-survivor patients were significantly older; this is particularly of note since we know that world’s population is aging progressively. In 2015, Roth et al. showed how global deaths from cardiovascular disease are increasing as a result of three important factors: population growth, the aging of populations, and epidemiologic changes in cardiovascular disease. For ischemic heart disease, the number of deaths increased by an estimated 41.7% from 1990 to 2013. Population ageing contributed to an estimated 52.5% increase in these deaths^10^. The Global Burden of Disease Study reported changes in mortality driven by population growth, population ageing, and changes in cause specific mortality rates^11^. We need to emphasize the fact that cardiovascular health and age interact in such a way that their contribution to patient outcome is critical in any clinical setting, as we could observe in COVID-19 patients.

In contrast to what was previously published^2,12^, in our cohort we did not find statistically significant association between typical CVRFs such as HT, DM, obesity, and dyslipidemia with mortality due to COVID-19. Interestingly, as reported by some authors^13,14^, we found that patients with lower GFR at admission estimated by the MDRD formula, were more prone to die, although GFR did not independently predict mortality. Even though CVRFs were not associated with mortality in our cohort, we still consider that the presence of these factors has an important role in cardiac function and global CV health, which in turn have been shown to be relevant regarding mortality due to COVID-19. In summary, we consider that CVRFs are probably a surrogate of impaired systolic and diastolic cardiac function in larger studies in which echo information is not available.

Concerning prior long-term treatment of this group of patients, it is worth noting that statins are postulated as a protective treatment against COVID-19^15^. The retrospective observational study published by Kuno et al. among 6095 patients with laboratory confirmed COVID-19 showed that, continuous use of statins was associated with lower in-hospital mortality compared to no statin use and discontinuation of statins^16^. The present work coincides with these observations, thus reinforcing the theory of a potential anti-inflammatory action of these agents that would favor the clinical course of the disease.

During the early weeks of the pandemic health contingency, it was postulated that treatment with drugs such as ACEI or ARA-2, would worsen the prognosis of patients^17^. However, no subsequent clinical study has reported an increase in mortality in patients treated with these drugs^18^, and even greater survival was described in patients treated with ARA-2^16^. Once again, our results follow the previous literature, reinforcing the theory that treatment with inhibitors of the Renin–Angiotensin–Aldosterone-System does not increase lethality in patients with SARS-CoV-2 infection.

In addition, it has been argued that COVID-19 enhances a prothrombotic state and several articles have reported an increase incidence of thromboembolic complications^19^. Despite the fact that some registries have shown a better prognosis in those patients undergoing anticoagulant therapy in the acute phase of COVID-19^20^, the evidence is insufficient as to recommend it in all cases of infection. Our paper is consistent with the above findings and describes a higher survival among patients being treated with anticoagulants, either with vitamin K inhibitors or with LMWH prior to and concomitantly with the start of hospitalization. Regarding DOACs therapy, there was no significant difference in terms of survival. Nevertheless, the prevalence of treatment with these was rather low (8.1%), making it difficult to demonstrate a statistically relevant difference.

From the echocardiographic approach, several studies reviewed the clinical significance of acute cardiac injury and the cytokine storm resulting in LV systolic dysfunction related to SARS-CoV-2 infection^21,22^. Our group was focused on the relationship between the prior state of cardiac function and the clinical prognosis.

According to recent investigations, myocardial injury in the clinical setting of COVID-19, previously defined by Kini et al.^23^, was reported in 20–30% of patients admitted to hospital and was associated with a poorer prognosis and higher mortality (50–60%). Such myocardial injury is particularly prevalent in more severely infected individuals and increases the likelihood of acute respiratory distress syndrome (ARDS), mechanical ventilation requirement, arrhythmias, renal failure and thrombotic events^24^. However, none of these studies provided information on previous ventricular function of the patients who developed myocardial damage and the associated complications mentioned above.

Many other cardiac COVID-19 related complications diagnosed by echocardiography have been described^25^, most notably pericardial effusion following pericarditis, myocarditis, right ventricular dysfunction resulting from pulmonary hypertension, and even cases of Tako-Tsubo syndrome^21^. Some authors highlight the importance of echocardiographic assessment of the RV, particularly prior to the initiation of invasive mechanical ventilation, given the negative effect of maintaining a high PEEP in patients with right ventricular dysfunction^25^. Right ventricular systolic function parameters seem to be relevant in terms of predicting clinical deterioration during admission^4–6^; it has been reported that the most common abnormal echocardiographic pattern among deteriorating patients (up to 50%) was RV dilatation and dysfunction associated with shortened acceleration time^4^. Li et al., reported that RV longitudinal strain (LS) in the lowest tercile (− 10.3 to − 20.5%) of their cohort had higher mortality during the acute phase of the disease; moreover, the best cutoff value for RVLS to predict outcome in this study was − 23%^26^. In our cohort, no statistically significant differences were found in any of the routinely used parameters for the assessment of right ventricular function (TAPSE, S′, CAF) and these do not seem to have played a relevant role in the clinical outcome of SARS-CoV-2 infection. As for longitudinal strain we couldn’t perform the analysis on a sufficient number of patients to assess its impact on outcome.

Our research has shown, as well, a significant correlation between diastolic dysfunction (DD) prior to admission and mortality during the stay, with differences between groups in the mitral E/A ratio and in tissue Doppler E/e′ ratio. DD is known to have relevance in systemic diseases in which exist inflammation and fibrous tissue formation such as systemic sclerosis and other chronic processes such as cirrhosis or renal failure. Tennoe et al. described that DD is associated with higher mortality among patients with systemic sclerosis and exceeds pulmonary hypertension with respect to predicting mortality^27^. Liang et al. described the close link between chronic kidney disease progression and DD; together with systolic dysfunction it confers a higher mortality risk in patients with stages 3 to 5^14^. DD was shown as well to be associated with outcome in patients with decompensated cirrhosis; particularly an E/e′ ratio > 10 was independently associated with higher MELD score and mortality^28^. Regarding to SARS-Cov-2 infection, Skezely et al.^4^ documented that left ventricular DD was present in up to 16% of patients admitted with COVID-19 and, particularly, they found a significant association between mortality and elevated E/e′ ratio. The results in our study show a similar pattern in terms of outcome for patients who had DD prior to COVID-19 diagnosis.

As for systolic function, in the study from Skezely et al. the patients' LVEF was normal in 90% of cases, only 2 patients (2%) had a documented reduced LVEF in a previous echocardiographic examination. Low LVEF at baseline assessment proved to be a predictor of clinical deterioration and mortality during admission^4^. Several other studies have found LVEF to be a predictor of mortality in patients admitted for COVID-19. Diaz et al. reported that LVEF was associated independently with mortality within 60-days of admission^29^. Barman et al. reported that patients with severe COVID-19 had lower LVEF than the non-severe patients. Additionally, Baycan et al. documented a significantly lower global longitudinal strain in patients with severe disease compared to the non-severe group and controls^30,31^. Assessment of LVEF in all of these studies was performed during the acute phase of the disease, and previous heart function status either remained unknown or was not taken into consideration when determining the clinical prognosis, regardless of whether or not there was cardiac damage during the infection.

Only a few studies had evaluated also the potential impact of LVEF prior to infection on COVID-19 outcomes. A large multicenter retrospective cohort including 8920 patients which analyzed the impact of a previous history of heart failure on in-hospital mortality during the first wave of the disease found that heart failure was associated with a higher risk of mortality. 335 of these patients had LVEF information available, and in this subgroup, LVEF lower than 40% was associated with the highest risk^32^. On the contrary, another retrospective study of 396 patients with an echo prior to admission due to COVID-19 did not find a significant association between LVEF and outcomes^33^. Important to note, in both studies, no other echo information was evaluated, and, thus, they had the limitation of excluding diastolic function and its potential interaction with heart failure on in-hospital mortality.

In our study, echocardiographic evaluation corresponds to the 12 months prior to admission for COVID-19, and plenty of other echocardiographic parameters have been assessed, including the aforementioned DD parameters. Most of our cohort had a considered normal LVEF mean value prior to admission. However, we have found that even patients with LVEF considered as normal, have high risk of mortality due to COVID-19, particularly if LVEF is < 60%, which is considered an accepted normal value^7^.

In our group of patients hospitalized due to COVID-19, the combination of systolic and diastolic function, represented by baseline LVEF and average E/E′ ratio, provide additional prognostic information and higher discriminative performance over demographic data such as age and gender.

Since emerging infectious diseases such as the COVID-19 are expected to occur and population ageing conducts to increases in total deaths for most leading causes, we need a full understanding of the burden and effect that, both, cardiac status and aging, could have on the clinical outcome of these emergent diseases.

### Limitations

The main limitation of the current study lies in the fact that it is a single-center, retrospective cohort study with a rather small sample size. In addition, it was not possible to follow up the patients who survived the event owing to the epidemiological context and the health authorities' restrictions. The study population mostly included non-ICU hospitalized patients, so interpretation of our findings is limited to this patient population. It is also important to note that the percentage of patients admitted to ICU may not reflect severity of the disease. During the first wave, with the massive admission of patients, we were struggling with limited ICU capacity and resources.

Furthermore, some parameters, such as the LV and RV global longitudinal strain, both known to be more robust and sensitive prognostic markers for patients affected with COVID-19.

## Conclusions

Age, male gender, anticoagulation status and prior cardiac function were found to be independent predictors of in-hospital mortality in our cohort of patients admitted for COVID-19. LVEF as an assessment of left ventricular systolic function and the E/e′ ratio as an indicator of diastolic function, significantly improved the discriminative power of our model for in-hospital mortality, compared to clinical factors alone.

## Data Availability

The datasets used and/or analyzed during the current study are available from the corresponding author on reasonable request.
